# Extracting the Energy Sensitivity of Charge Carrier Transport and Scattering

**DOI:** 10.1038/s41598-018-28288-y

**Published:** 2018-07-13

**Authors:** Shuang Tang

**Affiliations:** College of Engineering, State University of New York, Polytechnic Institute, Albany, New York 12203 USA

**Keywords:** Thermoelectric devices and materials, Thermoelectrics

## Abstract

It is a challenge to extract the energy sensitivity of charge carriers’ transport and scattering from experimental data, although a theoretical estimation in which the existing scattering mechanism(s) are preliminarily assumed can be easily done. To tackle this problem, we have developed a method to experimentally determine the energy sensitivities, which can then serve as an important statistical measurement to further understand the collective behaviors of multi-carrier transport systems. This method is validated using a graphene system at different temperatures. Further, we demonstrate the application of this method to other two-dimensional (2D) materials as a guide for future experimental work on the optimization of materials performance for electronic components, Peltier coolers, thermoelectricity generators, thermocouples, thermopiles, electrical converters and other conductivity and/or Seebeck-effect-related sensors.

## Introduction

The study of charge carrier transport focuses on the collective behaviors of electrons and holes in both real space and momentum space under external force fields, especially electrical fields. Quantum transport can be observed in single-electron devices^1–6^ and in highly ordered mesoscopic systems at cryogenic temperatures^7–13^, but the statistical transport behavior of multiple carriers for most material systems in modern applications must be described by diffusive models, such as the Boltzmann equations^14–27^.

Various scattering mechanisms may exist in diffusive transport^10,28–37^. Generally, the scattering strength and transport strength are inversely proportional to each other. By using an electrical conductivity (*σ*) matching method^10,33,38–48^ or observations of fast laser-assisted photon-electron interactions^49–54^, the average scattering time of carriers can be obtained and used to estimate the scattering strength. However, scattering and transport are also sensitive to the carrier energy. Therefore, if we can develop a method to extract the energy sensitivities of both carrier scattering and transport from experiments with real materials, we will have more instructive information on how to improve diffusive-transport-related applications in electronics, mechatronics and thermoelectronics.

The advancement of novel layered two-dimensional (2D) materials, including graphene^41,42,55–65^, transition metal dichalcogenide (TMD) layers^66^, and black phosphorene (BP)^67–71^, has provided a convenient testing platform for developing an energy sensitivity extraction method. These materials have simple band structures, where only a single valley or a few degenerate valleys are involved in transport^72–81^. Further, the Fermi levels and carrier concentrations of these materials can be efficiently tuned^67,70,82–96^. The transport behavior of graphene carriers has been intensively studied^10,33,38–48,97^ and can serve as a reliable system for testing new methods. Most importantly, this new method could also be used to improve the fundamental understanding of TMD and BP layers.

In this paper, we first define the energy sensitivities of carrier scattering and carrier transport within the diffusive transport regime. We then develop a new method to detect such energy sensitivities and test it in a graphene system at different temperatures. After that, we show how to use this method in other novel 2D material systems. Doing so will open a wide range of potential applications in transport-related research. For example, the method can provide information that can be used to analyze the major scattering sources in materials for conductivity improvement, to help engineer the types and concentrations of scattering centers to enhance the efficiency of Peltier cooling and/or thermoelectricity generating, and to help design various sensors, including thermocouples^98^, thermopiles^98^, electrical converters^99,100^, vacuum sensors^101,102^, flow sensors^103,104^, radiation sensors^105,106^, and special chemical sensors^107,108^, using the Seebeck effect.

## Method

Within the diffusive transport regime, the relation between the carrier scattering and the transport, including the electrical conductivity (*σ*) and the Seebeck coefficient (*S*)^35,109^, can be described by the Boltzmann equations. Rigorous solutions that include the elastic and inelastic scatterings in both degenerate and non-degenerate cases can be obtained by iterative approaches, e.g., Rode’s method^36,110–118^ (See the supplementary materials for further explanations). It is well known that under the relaxation time approximation, the transport properties can generally be approximated as

1 $$\sigma ={q}^{2}\int (-\frac{\partial {f}_{0}}{\partial \varepsilon })\Xi (\varepsilon )d\varepsilon $$

2 $$S=\frac{{k}_{B}}{q}\frac{\int (-\frac{\partial {f}_{0}}{\partial \varepsilon })\Xi (\varepsilon )(\varepsilon -{\varepsilon }_{f})d\varepsilon }{\int (-\frac{\partial {f}_{0}}{\partial \varepsilon })\Xi (\varepsilon )d\varepsilon }$$

3 $$\Xi (\varepsilon )=\tau (\varepsilon )D(\varepsilon ){\langle {v}^{2}\rangle }_{\varepsilon }$$

where *q* is the charge per carrier, *T* is the absolute temperature, *k*_*B*_ is the Boltzmann constant, *ε* is the reduced carrier energy (i.e., *ε* = *E/k*_*B*_*T*), *ε*_*f*_ is the reduced Fermi level, *f*_0_ is the Fermi-Dirac distribution, and Ξ (*ε*), *τ*(*ε*), and *D*(*ε*) are the transport distribution, scattering time and electronic density of states as a function of *ε*^31^, respectively. Further, *v* is the group velocity of the carriers, and the operator ${\langle \cdot \rangle }_{\varepsilon }$ stands for the mean value on the constant energy surface. Here, ${\langle {v}^{2}\rangle }_{\varepsilon }\propto {\varepsilon }^{r}$ and *r* = 0 (*r* = 1) for a linear (parabolic) band. $D(\varepsilon )\propto {\varepsilon }^{l}$ and *l* = 1 (*l* = 0) for a 2D linear (parabolic) band.

We can see that the transport properties are ultimately determined by the transport distribution Ξ (*ε*). Without loss of generality, the strength of transport at an arbitrary energy *ε* = *ε*_0_ can be characterized by *θ* = Ξ (*ε*_0_). The energy sensitivity of transport can then be characterized by

4 $$s{|}_{{\varepsilon }_{0}}={\left.\frac{d\Xi /\Xi }{d\varepsilon /\varepsilon }\right|}_{{\varepsilon }_{0}}.$$

Generally, the energy sensitivity *s* is a function of the carrier energy, temperature and carrier valley. For a single carrier, it is a specific value, but for the collective behavior of multiple carriers, it is a statistical measurement of the whole system. These statistical measurements are commonly used for diffusive transport; e.g., the carrier mobility is a statistical measurement of how mobile the carriers behave under an electrical field. Similarly, the strength of scattering at an arbitrary *ε* = *ε*_*0*_ can be characterized by *ξ* = 1/*τ*(*ε*_*0*_), and the energy sensitivity of scattering can be characterized by

5 $$j{|}_{{\varepsilon }_{0}}=-{\left.\frac{d\zeta /\zeta }{d\varepsilon /\varepsilon }\right|}_{{\varepsilon }_{0}}.$$

These two energy sensitivities (*s* and *j*) can be connected by Equation (3), e.g., *s* = *j* + 1, for a specific 2D band valley.

Intuitively, it is reasonable to expect that the same crowds of point defects will have weaker scattering effects (and, hence, stronger transport) to the higher energy carriers near a specific carrier pocket. The carrier energy sensitivity defined here can be then used to quantitatively evaluate the percent change in scattering/transport with respect to the percent change in carrier energy. This is unlike a traditional TEP model, where a presumed constant is used to simplify the model and calculations. Because TEP is not sensitive to transport data, the fit can generally be accepted; therefore, it does not provide a perspective on how the carriers’ scattering and transport in different systems react to changes in the carrier energy. As we will prove below, the concept of carrier energy sensitivity is not a presumed constant but a physical quantity that can vary with the carriers, systems and scattering mechanisms. It is also sensitive to the maximum Seebeck coefficient and can therefore be detected with a relatively high accuracy and used to infer the scattering mechanisms.

The rigorous Seebeck coefficients can be calculated through iterative approaches, e.g., Rode’s method^36,110–118^. Therefore, both elastic and inelastic scattering can be considered based on Equations (S14) and (S15).

Through our theoretical derivations, we have found that for a given band structure, the Seebeck coefficients for a specific Fermi level (i.e., carrier concentration) increase monotonically with the energy sensitivity of transport (*s*). Further, we have discovered that the maximum values of the Seebeck coefficients (*S*_*m*_) form a near-linear relation with the energy sensitivity, i.e.,

6 $${S}_{m}\approx s\frac{0.94\,{k}_{B}}{q}+{S}_{0},$$

where *S*_0_ is a band-structure-specified quantity. A software application to observe this relation in a general case is written using Matlab for this paper. These findings imply that once we have measured the *S*_*m*_ values of a system at a specific temperature, we can deduce the energy sensitivity of transport *s* (and, thus, the energy sensitivity of scattering *j*) at this temperature. Generally, there will be only one local optimal Seebeck coefficient corresponding to each band valley. Therefore, for a materials system with single- or degenerate-valley transport, such as 2D layered materials, the valley-specific energy sensitivity can be deduced directly. For a material with non-degenerate-valley transport, multiple local optimums of the Seebeck coefficient should be considered for the deduction. Once the energy sensitivity of transport (*s*) is measured with this method, the energy sensitivity of scattering (*j*) can be naturally obtained. For a material with a single scattering channel, *j* is simply the channel-specific energy sensitivity. For a case with multiple scattering channels, *j* is the effective energy sensitivity that represents a weighted average of all existing channels, which is also a statistical measurement.

## Method Validation

**Figure 1:**
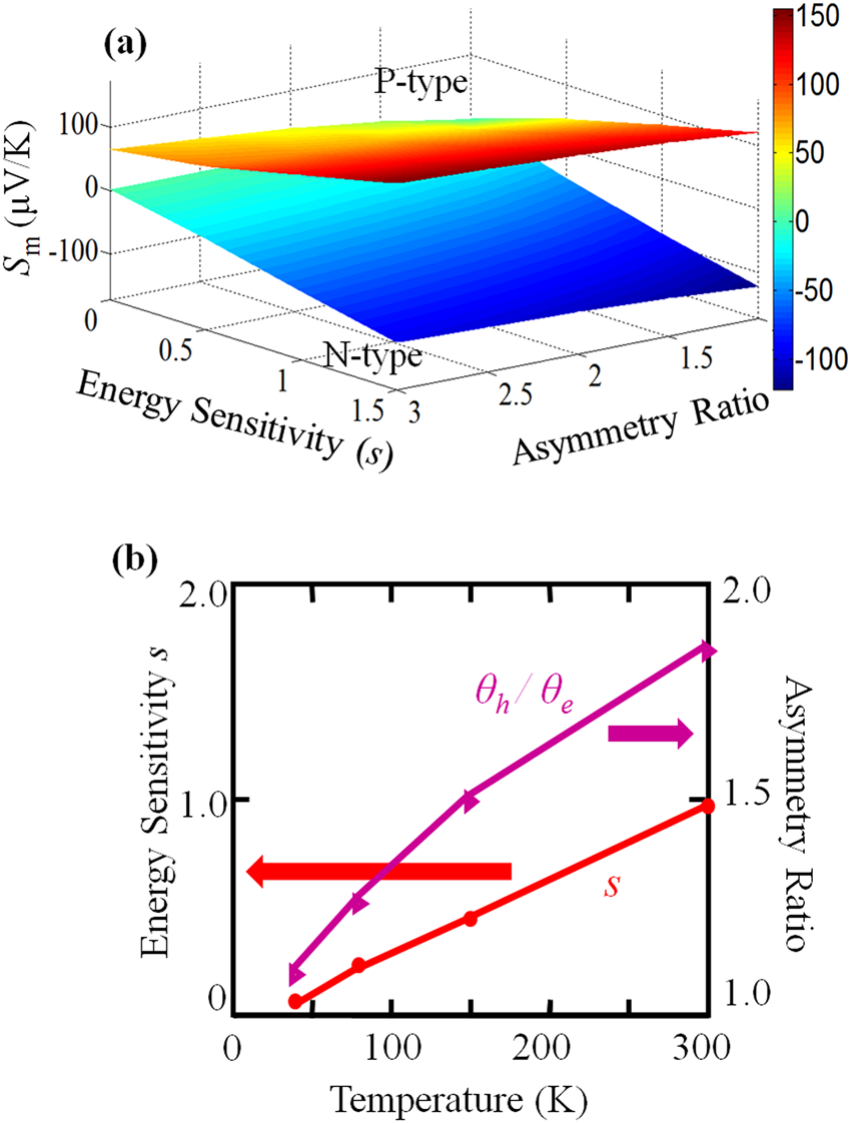
The new method was validated using a graphene system. (**a**) Plots of the optimal Seebeck coefficient (*S*_*m*_) of graphene are presented for both *N*-type and *P*-type regimes, as a function of energy sensitivity (*s*) and the asymmetry ratio of scattering strength *θ*_*h*_/*θ*_*e*_ at 300 K. (**b**) The results of the energy sensitivity (*s*) and asymmetry ratio (*θ*_*h*_/*θ*_*e*_) of carrier scatterings near the band valley in graphene correspond to the data in Table 1.

Now, we test this method in a graphene system. With the development of nanotechnology for device fabrication, the measurement of Seebeck coefficient (*S*) as a function of Fermi level is now available^82–91^. The carriers that contribute to electronic transport come mainly from the two isotropic Dirac cones, whose apexes are near the Fermi level in the first Brillouin zone. The rigorous Seebeck coefficient can be calculated using the iterative approaches method^36,110–118^. Therefore, both the elastic and inelastic scatterings can be considered based on Equations (S14) and (S15). For convenience of calculation, we can first calculate a map of *S*_*m*_ as a function of *s* and γ = *θ*_*h*_/*θ*_*e*_ for both the *P*- and *N*-type regimes. This *S*_*m*_ map will vary with temperature. We illustrated an example at *T* = 300 K in Fig. 1(a). Then, by matching the measured values of *S*_*m*_ to this map, we can obtain a single solution set for *s* and γ = *θ*_*h*_/*θ*_*e*_ at each temperature. The *S*_*m*_ values of graphene at different temperatures have been measured in previous reports^82–91^. Using ref.^84^ as an example, where a graphene system on a SiO_2_ substrate is measured using a gated thermoelectric device with Fermi level tuning. The configuration used for the experimental setup is explained in Fig. 1 of ref.^84^. Their data are summarized in Table 1. We then determined the values of the energy sensitivity of transport *s* and the asymmetry ratio γ = *θ*_*h*_/*θ*_*e*_ at each temperature, according to Table 1. Our results are shown in Fig. 1(b).

**Table 1:** Measured Optimal Seebeck Coefficient^84^ for the Graphene on a SiO_2_ substrate^84^.

Temperature (K)	300	150	80	40
*P*-type *S*_*m*_ (μV/K)	92.52	57.94	33.64	14.95
*N*-type *S*_*m*_ (μV/K)	−59.81	−39.25	−24.30	−10.28

From Fig. 1(b), we see that the energy sensitivity *s* changes significantly with temperature, which implies that the effective carrier scattering mechanism is very temperature-sensitive. When approaching the low temperature end, the energy-sensitivity behaves as *s* → 0, which implies that ${\tau }^{-1}\to {\tau }^{-1}\propto D(\varepsilon ),$ and the scattering is negatively sensitive to the energy of carriers. When approaching high temperatures end, the sensitivity behaves as *s* → 1, which implies that *τ* tends to become constant over a different range of carrier energies. This information about *τ*(*ε*) can be explained by the scattering mechanism(s) of graphene carriers. In the low temperature range, the dominant scattering mechanisms should be acoustic phonon scattering and short-range disorder scattering, such as surface roughness^119–121^, point defects and vacancies^122^, that are intrinsically formed by the carbon atoms within the graphene sheet. For these scattering mechanisms, the scattering time *τ*(*ε*) will be inversely proportional to the density of states *D*(*ε*)^120,123^. Equation (3) then suggests that *s* → 0. The small deviation of *s* from 0 might be due to minor scattering mechanism(s) or may occur because the dispersion relation can be disturbed from linearity to a certain extent near the apex of Dirac cones^124–126^. At elevated temperatures, the inelastic scattering due to the optical phonons becomes important. The scattering time for such inelastic scattering is usually constant over a range of carrier energies, i.e., *j* = 0, either by the rigorous solution or the relaxation time approximation of the Boltzmann equation, which is consistent with the results. On the other hand, at elevated temperatures, the carrier scattering mechanism(s) induce higher values of *j*, which then become(s) important; e.g., thermal ripple scattering^119,127–130^ will have *j* = 2. This high-*j* scattering now coexists with and compensates for the low-*j* scatterings, e.g., the acoustic phonon scattering (*j* = −1), which makes the statistical *j* → 0, i.e., *s* → 1.

**Figure 2:**
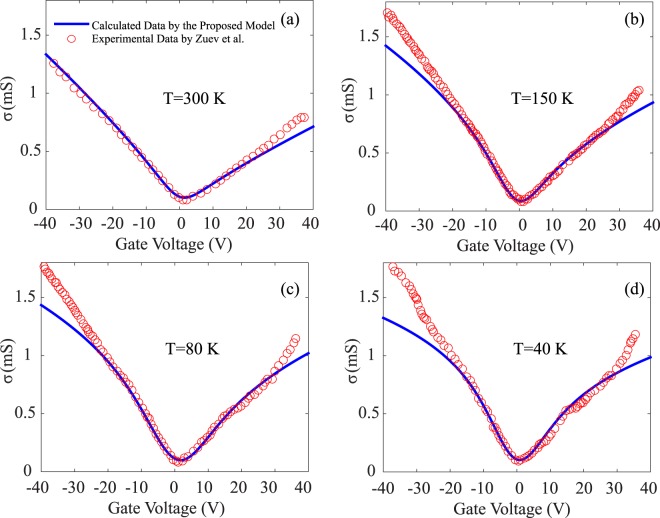
Comparison between the electrical conductivity (*σ*) data at (**a**) 300 K, (**b**) 150 K, (**c**) 80 K and (**d**) 40 K, as determined by the proposed model (blue lines) and experimental measurement (red circles)^84^. It can be seen that the model is generally quite consistent with the experimental data.

Another important trend is shown in Fig. 1(b), where the asymmetry ratio *θ*_*h*_/*θ*_*e*_ increases with temperature, but the electrons and the holes are close to being symmetric at temperatures as low as 40 K. This is consistent with the previous report that electrons and holes are asymmetric when they transported in graphene-related systems^131^, even though they are symmetric in the dispersion relation. Furthermore, the scattering strengths for electrons and holes deduced from this new approach are ${\xi }_{e}=0.5\times {10}^{14}{\text{s}}^{-1}$ and ${\xi }_{h}=2.63\times {10}^{13}{\text{s}}^{-1}$ at 300 K, respectively. Further, in our above calculations for graphene, we assume that the electronic transport comes from only the carriers near the apex of the Dirac cones, and ignoring the contributions from higher energy carriers. To evaluate how this deviates from reality, we compared the electrical conductivity data from the above model to experimental measurements at different temperatures, as shown in Fig. 2. Although we are now considering only the two band valleys of the Dirac cone, the modeled data are already quite consistent with the experimental measurements. The deviations at higher energy occur because more bands are contributing to the transport.

**Figure 3:**
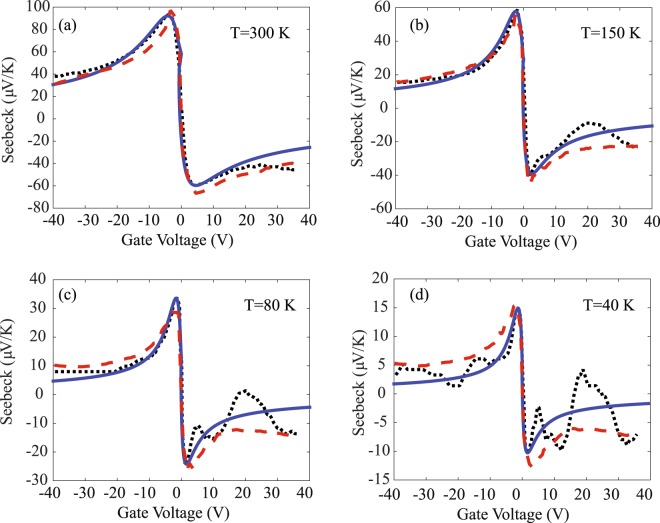
Comparison of the Seebeck coefficient data from experimental measurements (dotted lines), the Mott’s relation (dashed lines) and the proposed model (solid lines). It can be seen that the proposed model is generally more accurate than Mott’s relation for data near the Seebeck peak, especially at lower temperatures.

Traditionally, Mott’s relation is used to model the Seebeck coefficient, where

7 $${S}_{Mott}({E}_{f})=-\,\frac{{\pi }^{2}}{3q{k}_{B}\sigma }{(\frac{\partial \sigma }{\partial \varepsilon })}_{{\varepsilon }_{f}},$$

which suggests that the Seebeck coefficient can be simply obtained from the electrical conductivity data. However, our theoretical derivation has noted that the Mott’s relation is only suitable to obtain Seebeck coefficients that are far from the optimal points. To further demonstrate this, we have compared the Seebeck coefficient values from the experiments, the Mott’s relation, and our proposed model, as shown in Fig. 3. In the graphene system, the proposed method is generally better than Mott’s relation for Seebeck data near the peak, and the advantage of the proposed method becomes increasingly obvious at lower temperatures.

## Discussion

**Figure 4:**
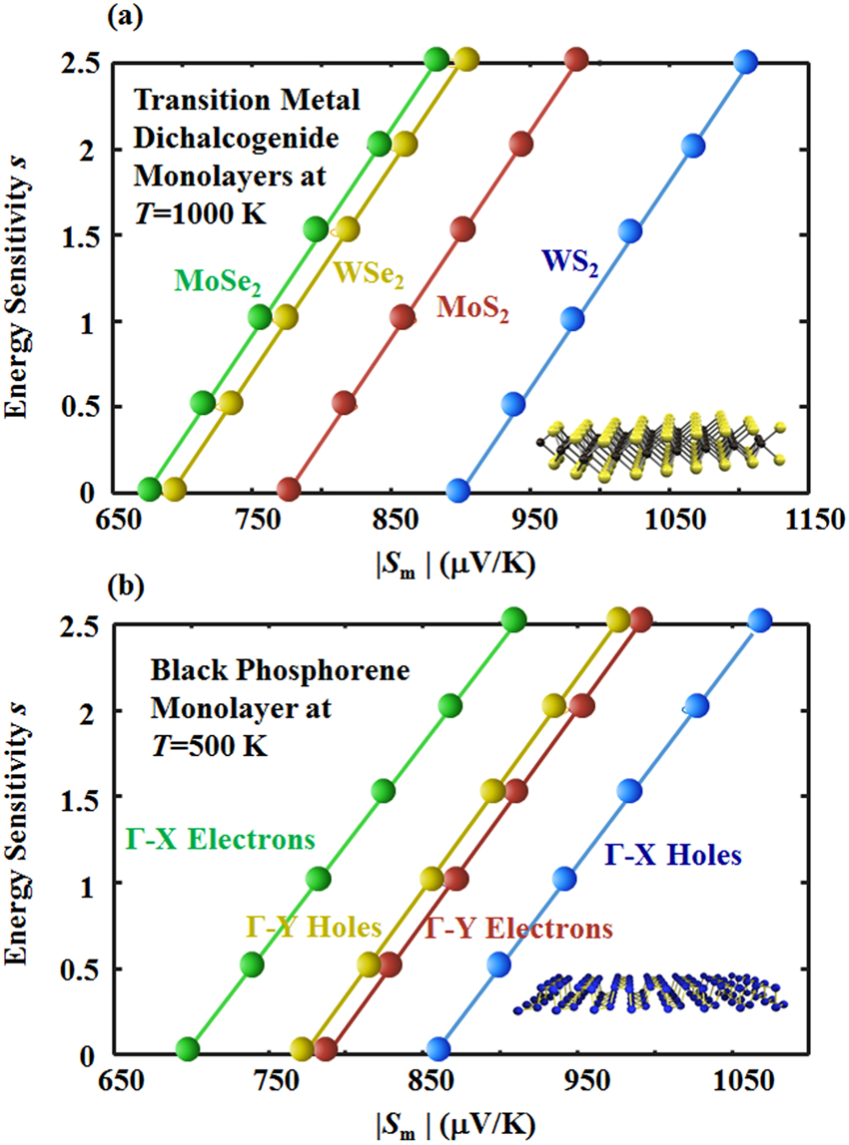
How the optimal Seebeck coefficient (*S*_*m*_) can be used to infer the energy sensitivity of transport (*s*) for various (**a**) TMD monolayers and (**b**) black phosphorene monolayer. The filled dots show calculated data, and the solid curves show a fitted linear relation from these data for each type of 2D materials system. The band valleys of the electrons and holes in black phosphorene are anisotropic, and both the Γ-X and Γ-Y directions are illustrated here. The general approach for modeling an anisotropic system is discussed in the supplementary materials, and details can be found in ref.^109^.

Now, we use the new method to study other layered 2D materials. TMD monolayers are another class of 2D materials^73–81^, where the transport involved band valleys at the *K* and *K’* points are degenerate for the conduction side and valence side. The electrons and holes are parabolically dispersed at the band edges. Figure 4(a) shows the results for how the energy sensitivity of transport is derived from the optimal values of the Seebeck coefficient with our new method. The one-to-one correspondence and linear relation still hold for various TMD monolayer systems. BP is another novel layered 2D material with parabolically dispersed band edges located at the *Γ* point in the Brillouin zone^70,132–135^. Figure 4(b) shows how the energy sensitivity of transport can be solved from the optimal values of the Seebeck coefficient for a single layer of BP. Both the Γ-X and Γ-Y directions are exhibited for anisotropic transport in BP^136–138^. Details on modeling an anisotropic system are given in the supplementary materials and in ref.^109^.

Most of these novel layered 2D materials have single-valley scattering and are therefore an ideal starting point for this new method. For a band structure that involves multiple non-degenerate band valleys in transport, each valley will induce a peak or kink in the Seebeck coefficient vs. Fermi level curve. Therefore, to extend the new method to such a general system, the non-primary peaks or kinks of the Seebeck coefficient will be used to solve the values of energy sensitivity (*s*).

Further, we know that for ballistic transport^139–142^,

8 $$\sigma ={q}^{2}\int (-\frac{\partial {f}_{0}}{\partial \varepsilon }){\bf{T}}(\varepsilon )d\varepsilon $$

9 $$S=\frac{{k}_{B}}{q}\frac{\int (-\frac{\partial {f}_{0}}{\partial \varepsilon }){\bf{T}}(\varepsilon )(\varepsilon -{\varepsilon }_{f})d\varepsilon }{\int (-\frac{\partial {f}_{0}}{\partial \varepsilon }){\bf{T}}(\varepsilon )d\varepsilon }$$

where **T** is the total transmission probability function. Equations (1) and (2) are exactly the same as Equations (8) and (9), except for the definitions of Ξ and **T**. Therefore, we can use Ξ as a broader function: Ξ serves as the transport distribution function for diffusive channels and the total transmission probability for ballistic channels, and it can describe the combination of multiple mixed channels. Therefore, the energy sensitivity can be measured using this more general function, Ξ .

## Conclusion

Based on our theoretical derivations and numerical validations, we have proposed that the optimum values of the Seebeck coefficient can be used as a new tool to extract the carrier energy sensitivity of transport and scattering from experimental data. This statistical measurement can provide us with deeper information to improve applications related to diffusive transfer in semiconducting and metallic materials. We have validated this new method using a graphene system at different temperatures. Then, we have shown how to use the validated method for other layered 2D materials, including various transition metal dichalcogenide layers and a black phosphorene layer. This will allow a wide range of potential applications in transport-related research, including electronic devices, Peltier coolers, thermoelectricity generators, thermocouples, thermopiles, electrical converters, and other sensors using the Seebeck effect, e.g., vacuum sensors, flow sensors, radiation sensors, and chemical sensors.

## Electronic supplementary material

Supplementary Materials
