# Supplementary material for: Extracting the Energy Sensitivity of Charge Carrier Transport and Scattering
Source: Sci Rep. 2018 Jul 13;8:10597. doi: 10.1038/s41598-018-28288-y (PMC6045660; doi:10.1038/s41598-018-28288-y)
Supplement: Supplementary file 1 — Supplementary Materials [file 41598_2018_28288_MOESM1_ESM.docx]

**Extracting the Energy-Sensitivity of Charge Carrier Transport and Scattering**

Shuang Tang*^a^

^a^ College of Engineering, State University of New York, Polytechnic Institute, Albany, New York 12203, USA. E-mail: [tangs1@sunyit.edu](mailto:tangs1@sunyit.edu)

**SUPPLEMENTARY MATERIALS**

**1. The derivation of one-to-one correspondence between the energy-sensitivity of transport and the optimal Seebeck coefficient**

From Equation (2), we have

$S\frac{q}{k_{B}}=(\frac{\int\left(-\frac{\partial f}{\partial\varepsilon}\right)\Xi\varepsilon d\varepsilon}{\int\left(-\frac{\partial f}{\partial\varepsilon}\right)\Xi d\varepsilon}-\varepsilon_{f})$. (S1)

In order to see how the Seebeck coefficient changes with the scattering order, we make a variant of *S* with respect to *j* on both sides of Equation (S1),

$\frac{\delta S}{\delta s}\frac{q}{k_{B}}=\frac{\int\left(-\frac{\partial f}{\partial\varepsilon}\right)\frac{\partial\Xi}{\partial s}\varepsilon d\varepsilon\int\left(-\frac{\partial f}{\partial\varepsilon}\right)\Xi d\varepsilon-\int\left(-\frac{\partial f}{\partial\varepsilon}\right)\Xi\varepsilon d\varepsilon\int\left(-\frac{\partial f}{\partial\varepsilon}\right)\frac{\partial\Xi}{\partial s}d\varepsilon}{\left[\int\left(-\frac{\partial f}{\partial\varepsilon}\right)\Xi d\varepsilon\right]^{2}}$. (S2)

By simplifying Equation (S2) by Equation (4), we have

$\frac{\delta S}{\delta s}\frac{q}{k_{B}}=\frac{\int\left(-\frac{\partial f}{\partial\varepsilon}\right)\Xi\ln\left(\varepsilon\right)\varepsilon d\varepsilon\int\left(-\frac{\partial f}{\partial\varepsilon}\right)\Xi d\varepsilon-\int\left(-\frac{\partial f}{\partial\varepsilon}\right)\Xi\varepsilon d\varepsilon\int\left(-\frac{\partial f}{\partial\varepsilon}\right)\Xi\ln\left(\varepsilon\right)d\varepsilon}{\left[\int\left(-\frac{\partial f}{\partial\varepsilon}\right)\Xi d\varepsilon\right]^{2}}$, (S3)

which can be rearranged to be

$\frac{\delta S}{\delta s}\frac{q}{k_{B}}=\frac{\int\left[\varepsilon-\frac{\int\varepsilon\left(-\frac{\partial f}{\partial\varepsilon}\right)\Xi d\varepsilon}{\int\left(-\frac{\partial f}{\partial\varepsilon}\right)\Xi d\varepsilon}\right]\left[\ln\left(\varepsilon\right)+1\right]\left(-\frac{\partial f}{\partial\varepsilon}\right)\Xi d\varepsilon}{\int\left(-\frac{\partial f}{\partial\varepsilon}\right)\Xi d\varepsilon}$. (S4)

Now we try to consider the term $\left(-\frac{\partial f}{\partial\varepsilon}\right)\Xi$ as a probability distribution as a function of $\varepsilon$, which we denote the normalized distribution as

$p\left(\varepsilon\right)=\frac{\left(-\frac{\partial f}{\partial\varepsilon}\right)\Xi}{\int\left(-\frac{\partial f}{\partial\varepsilon}\right)\Xi d\varepsilon}$. (S5)

Thus, the expression can be largely simplified from the understanding of probability theory. We will have,

$\left\langle\varepsilon\right\rangle=\int\varepsilon pd\varepsilon=\frac{\int\varepsilon\left(-\frac{\partial f}{\partial\varepsilon}\right)\Xi d\varepsilon}{\int\left(-\frac{\partial f}{\partial\varepsilon}\right)\Xi d\varepsilon}$, (S6)

and further,

$\frac{\delta S}{\delta s}=\int\left[\varepsilon-\left\langle\varepsilon\right\rangle\right]\left[\ln\varepsilon+1\right]pd\varepsilon=\int\left[\varepsilon-\left\langle\varepsilon\right\rangle\right]\ln\varepsilon pd\varepsilon=\left\langle\varepsilon\ln\varepsilon\right\rangle-\left\langle\varepsilon\right\rangle\left\langle\ln\varepsilon\right\rangle$. (S7)

This is naturally the expression for the covariance between and, i.e.

$Cov\left(\varepsilon,\ln\varepsilon\right)=\frac{q}{k_{B}}\frac{\delta S}{\delta s}$. (S8)

Because $\ln\varepsilon$ is a strictly increasing function of $\varepsilon$, the covariance between $\varepsilon$ and $\ln\varepsilon$ is always positive. Therefore, we have proved that *S* is also a strictly increasing function of *s*, at a specific $\varepsilon_{f}$. This implies that each value of *S* will correspond to a single value of *s*, so if we can measure the value of *S*, we will be able to detect the carrier energy-sensitivity of transport (*s*).

To further make this energy-sensitivity tool easy to use, we now try to prove that the energy-sensitivity of transport (*s*) has a near-linear relation with the optimal values of the Seebeck coefficient near the band edge. Since $\ln\varepsilon$is a strictly increasing function, so the more weight the probability distribution *p*(*ε*) has on the high ε end, the larger the covariance will be. This is to say that the fatter the *p*(*ε*) is, the larger Cov(*ε*, ln*ε*) will be. Near the band edge, we have

$\left(-\frac{\partial f}{\partial\varepsilon}\right)\Xi\to\frac{e^{\varepsilon}\Xi}{\left(e^{\varepsilon}+1\right)^{2}}$. (S9)

For the high *ε* end, *p* decrease with *ε* in the same order as Ξ/*e^ε^*. Therefore, the larger *s* is, the slower *p*(*ε*) will decay, and the larger Cov(*ε*, ln*ε*) will be. In other words, Cov(*ε*, ln*ε*) is also a function of *s*. We can then examine what Cov(*ε*, ln*ε*) will be within the possible range for *s*, which is typically 0~3 for a diffusive transport system. The result shows that Cov(*ε*, ln*ε*) takes values within the range between 0.91 and 0.97, when s is between 0 and 3. Therefore, we have approximately,

$\frac{\delta S}{\delta s}\approx\frac{k_{B}}{q}\cdot0.94$, (S10)

near the band edge, which leads to Equation (6).

**2. Why Mott’s relation cannot be employed here**

Mott's relation is widely used in calculations of the Seebeck coefficient from measured electrical conductivity ^[1-3](#_ENREF_1)^. We point out here that Mott's relation only captures the Seebeck coefficient corresponding to a Fermi level that is far away from the energy range where the Seebeck coefficient is optimized; it fails to capture the maximum Seebeck coefficient. Such discrepancy comes from the difference between Mott's relation and Equation (2). From the Boltzmann relation given by Equation (2), we have the Seebeck coefficient for a single valley as,

$S=\text{-}\frac{k_{B}}{q}\frac{\int\left(-\frac{\partial f_{0}}{\partial\varepsilon}\right)\left(\varepsilon-\varepsilon_{f}\right)\Xi d\varepsilon}{\int\left(-\frac{\partial f_{0}}{\partial\varepsilon}\right)\Xi d\varepsilon}$, (S11)

while Mott's relation [^1-3^](#_ENREF_1)is

$S_{Mott}\left(E_{f}\right)=-\frac{\pi^{2}}{3qk_{B}\sigma}\left(\frac{\partial\sigma}{\partial\varepsilon}\right)_{\varepsilon_{f}}$, (S12)

which gives the approximated Seebeck coefficient $S_{Mott}$ by

$S_{Mott}=\frac{k_{B}}{q}\frac{\int\left(-\frac{\partial f_{0}}{\partial\varepsilon}\right)\Xi\frac{\pi^{2}}{3}\tanh\left(\frac{\varepsilon-\varepsilon_{f}}{2}\right)d\varepsilon}{\int\left(-\frac{\partial f_{0}}{\partial\varepsilon}\right)\Xi d\varepsilon}$. (S13)

In essence, Mott's relation uses the hyperbolic tangent relation$\left(\pi^{2}/3\right)\tanh[\left(\varepsilon-\varepsilon_{f}\right)/2]$ to approximate the linear relation $\left(\varepsilon-\varepsilon_{f}\right)$, which can make a significant difference to the Seebeck coefficient determined over the Fermi level range where the maximum Seebeck coefficient occurs. Hence, we see that Mott's relation used widely in the thermoelectrics literature is only valid to model the Seebeck coefficient far away from the conditions where the Seebeck coefficient can be maximized.

**3. Characterization of the strength of transport**

The parameter *θ* is the transport distribution function Ξ when the carrier energy is equal to the thermal excitation *k_B_T*. The energy-sensitivity of Ξ can be used to compare the transport between carriers with different energies, however, if we want to compare between different transport distribution functions, e.g. Ξ_1_ and Ξ_2_, we need to compare them at the same energy level. Ξ at *E=0* is not a good energy level to be compared, because Ξ_1_ and Ξ_2_ will generally be equal to 0 for most cases. Therefore, the values of Ξ_1_ and Ξ_2_ when the carrier energy is equal to the thermal excitation *k_B_T* can serve as a natural measurement for such a comparison task. In the simplest cases, if Ξ_1_= Ξ_01_*ε^s^* and Ξ_2_= Ξ_02_*ε^s^*, we will have *θ*_1_= Ξ_01_ and *θ*_2_= Ξ_02_. This gives us a measure of how strong the transport is, besides the energy-sensitivity.

**4. Rigorous solution vs. relaxation time approximation**

To obtain the rigorous solution of Boltzmann equation, iterative methods can be used. In the Rode’s method [^4-12^](#_ENREF_4), the electron distribution can be written as [^13^](#_ENREF_13),

$f\left(\mathbf{k}\right)=f_{0}\left[\varepsilon\left(\mathbf{k}\right)\right]+xg\left(\mathbf{k}\right)$, (S14)

where *f* is the actual distribution of the electrons, and *f_0_* is the equilibrium Fermi-Dirac distribution, *x* is the cosine of the angle between the small driving force and **k**, *g*(**k**) is the perturbation to the distribution caused by the small driving force. The perturbation *g*(**k**) now include both the inelastic and elastic scatterings, as

, (S15)

where *S_i_* and *S_o_* are the scattering-in and scattering-out terms that can catch the inelastic scatterings, *χ_el_*(**k**) is the summation of all elastic scattering rates, including the acoustic phonon scattering, the ionized impurity scattering, the piezoelectric scattering, the deformation potential scattering and the dislocation scattering, etc. $\left(\frac{\partial f}{\partial z}\right)$ is the thermal driving force. *g*(**k**) is solved iteratively under the Rode’s method, where the more rigorous Seebeck coefficient and electrical conductivity can be obtained.

On the one hand, the rigorous solution based on the iterative method for Seebeck coefficient should be used to make the maps of Figure 1 (a)-(c), instead of Eq. (2). The further details can be found in Ref. [^10^](#_ENREF_10) and [^13^](#_ENREF_13). On the other hand, according to the comparison between Seebeck coefficient calculated by the Rode’s method and Eq. (2) for materials systems that have strong inelastic scatterings, we can see that though the calculated electrical conductivity can be essentially different, but the calculated Seebeck coefficient do not have significant difference, as discussed in Ref. [^10^](#_ENREF_10),[^13^](#_ENREF_13). This is why we think using Seebeck coefficient to interpret the energy dependence is more reliable than just using the electrical conductivity. For the inelastic scattering, the statistical energy-sensitivities are *j*=~0 and *s*=~1.

To sum up, for either the rigorous solution or the relaxation time approximation, the maximum Seebeck coefficient will form a bijective relation with the statistical energy-sensitivity of transport, so our new method can be used to obtain this energy-sensitivity. For most cases, the RTA can be used with tolerable error even when inelastic scattering are important. The average energy-sensitivity of scattering for full inelastic scattering will be *j*=0.

**5. Energy-sensitivity of scattering for different scattering mechanisms.**

|  | Linear Band Valley | Parabolic Band Valley |
| --- | --- | --- |
| Acoustic Phonon Scattering | -1 | 0 |
| Point Contact Scattering | -1 | -0.5 |
| Coulomb Interaction Scattering | 1 | 1.5 |
| Roughness Scattering | -1 | -0.5 |
| Thermal Ripples Scattering | 2 | 1 |
| Inelastic Scattering | 0 | 0 |

**6. The asymmetry ratio between the transport strength**

The asymmetry ratio we defined in the present work is not the ratio between the actual electrical conductivities of electrons and of holes, but the ratio between the transport distribution functions of electrons and of holes. Therefore, even for an *n*-type region, where the Fermi level is above the neutrality point. The concentration and conductivity of electrons are much larger than the concentration and conductivity of holes. However, the asymmetry ratio *γ* will still be finite, i.e.

$\gamma=\frac{\Xi_{h}}{\Xi_{e}}=\frac{\theta_{h}}{\theta_{e}}\neq\frac{\sigma_{h}}{\sigma_{e}}$. (S16)

In the case of high carrier concentration, where the graphene is in a highly degenerate situation, e.g. *n_h_*>>*n_e_* and *σ_h_*>>*σ_e_*, though *n_h_*/*n_e_*→∞ and *σ_h_*/*σ_e_*→∞, but γ will still be finite. This is because although

$\frac{n_{h}}{n_{e}}=\frac{\int_{holes} D\left(\varepsilon\right)\left(1-f\right)d\varepsilon}{\int_{electrons} D\left(\varepsilon\right)fd\varepsilon}$ (17)

and

$\frac{\sigma_{h}}{\sigma_{e}}=\frac{n_{h}\mu_{h}}{n_{e}\mu_{h}}$, (18)

but Ξ_1_ and Ξ_2_ are independent of the Fermi level.

1. **Anisotropic vs. Isotropic Materials Systems**

For an isotropic system, the transport function Ξ(*ε*) in Equation (3) can is adequate in describing transport for all different directions. For an anisotropic system, such as a black phosphorene (BP) system, the transport function in Equation (3) should be a tensor, i.e.

$\overleftrightarrow{\Xi}\left(\varepsilon\right)=\left(\begin{matrix} \Xi_{1}\left(\varepsilon\right) & \\ & \Xi_{2}\left(\varepsilon\right) \end{matrix}\right)=\left(\begin{matrix} \tau_{1}\left(\varepsilon\right)\left\langle v_{1}^{2}\right\rangle_{\varepsilon} & \\ & \tau_{2}\left(\varepsilon\right)\left\langle v_{1}^{2}\right\rangle_{\varepsilon} \end{matrix}\right)D\left(\varepsilon\right)$, (19)

where the subscript “1” and “2” stand for the different principal directions, such as the Γ-X direction and the Γ-Y direction in BP. This tensor equation can also be written for each component,respectively, i.e.

$\Xi_{1}\left(\varepsilon\right)=\tau_{1}\left(\varepsilon\right)D\left(\varepsilon\right)\left\langle v_{1}^{2}\right\rangle_{\varepsilon}$, (20)

and

$\Xi_{2}\left(\varepsilon\right)=\tau_{2}\left(\varepsilon\right)D\left(\varepsilon\right)\left\langle v_{2}^{2}\right\rangle_{\varepsilon}$. (21)

As we can see, each component has the same form as in Equation (3) except the subscript. For an isotropic case, the “1” direction and the “2” direction become the same. The principal components for the electrical conductivity tensor (*σ_1_* and *σ_2_*) and for the Seebeck coefficient tensor (*S_1_* and *S_2_*) can be calculated accordingly.

**REFERENCES:**

1 Ono, Y. & Taylor, P. Theory of the low-temperature Seebeck coefficient in dilute alloys. *Physical Review B* **22**, 1109 (1980).

2 Wei, P., Bao, W., Pu, Y., Lau, C. N. & Shi, J. Anomalous thermoelectric transport of Dirac particles in graphene. *Physical Review Letters* **102**, 166808 (2009).

3 Paul, B., Kumar, A. & Banerji, P. Embedded Ag-rich nanodots in PbTe: Enhancement of thermoelectric properties through energy filtering of the carriers. *Journal of Applied Physics* **108**, 064322 (2010).

4 Rode, D. *Semiconductors and Semimetals*. (Academic Press, 1975).

5 Miller, N. *et al.* Effect of charged dislocation scattering on electrical and electrothermal transport in n-type InN. *Phys. Rev. B* **84**, 075315 (2011).

6 Rode, D. Electron mobility in direct-gap polar semiconductors. *Phys. Rev. B* **2**, 1012 (1970).

7 Ng, G., Vasileska, D. & Schroder, D. Calculation of the electron Hall mobility and Hall scattering factor in 6H-SiC. *J. Appl. Phys.* **106**, 053719 (2009).

8 Lundstrom, M. *Fundamentals of carrier transport*. (Cambridge University Press, 2009).

9 Ferry, D. *Semiconductor transport*. (CRC Press, 2000).

10 Ramu, A. T. *et al.* Rigorous calculation of the Seebeck coefficient and mobility of thermoelectric materials. *J. Appl. Phys.* **107**, 083707 (2010).

11 Ramu, A. T. *et al.* Thermoelectric transport in the coupled valence-band model. *J. Appl. Phys.* **109**, 033704 (2011).

12 Ramu, A. T. & Bowers, J. E. The impact of commonly used approximations on the computation of the Seebeck coefficient and mobility of polar semiconductors. *Appl. Phys. Lett.* **101**, 173905 (2012).

13 Faghaninia, A., Ager III, J. W. & Lo, C. S. ab initio Electronic Transport Model with Explicit Solution to the Linearized Boltzmann Transport Equation. *Phys. Rev. B* **91**, 235123 (2015).
